# Predictors of Natural and Unnatural Mortality among Patients with Personality Disorder: Evidence from a Large UK Case Register

**DOI:** 10.1371/journal.pone.0100979

**Published:** 2014-07-07

**Authors:** Marcella Lei-Yee Fok, Robert Stewart, Richard D. Hayes, Paul Moran

**Affiliations:** 1 King's College London, King's Health Partners, Dept of Health Service and Population Research, Institute of Psychiatry, London, United Kingdom; 2 King's College London, King's Health Partners, Dept of Psychological Medicine, Institute of Psychiatry, London, United Kingdom; Nathan Kline Institute and New York University School of Medicine, United States of America

## Abstract

**Background:**

People with personality disorder have reduced life expectancy, yet, within this population, little is known about the clinical predictors of natural and unnatural deaths. We set out to investigate this, using a large cohort of secondary mental health patients with personality disorder.

**Methods:**

We identified patients with an ICD-10 diagnosis of personality disorder, aged ≥15 years in a large secondary mental healthcare case register. The case register was linked to national mortality tracing. Using Cox regression, we modelled the effect of a number of pre-specified clinical variables on all-cause, natural cause and unnatural cause mortality.

**Findings:**

2,440 patients were identified. Eighty-five deaths (3.5% of cohort) occurred over a 5-year observation period, of which over 50% were from natural causes. All-cause mortality was associated with alcohol or drug use (adjusted Hazard Ratio [aHR] 2.3; 95% CI 1.3–4.1), physical illness (aHR 1.9; 95% CI 1.0–3.6), and functional impairment (aHR 1.9; 95% CI 1.0–3.6). Natural cause mortality was associated with mild problems of alcohol or drug use (aHR 3.4; 95% CI 1.5–7.4), and physical illness (aHR 2.4; 95% CI 1.0–5.6). Unnatural cause mortality was associated only with severe alcohol or drug use (aHR 3.1; 95% CI 1.3–7.3).

**Interpretation:**

Alcohol and drug use, physical illness, and functional impairment are predictors of mortality in individuals with personality disorder. Clinicians should be aware of the existence of problems in these domains, even at mild levels, when assessing the needs of patients with personality disorder.

## Introduction

Personality disorders (PD) present a considerable health problem globally. They are highly prevalent mental disorders, affecting up to 10% of community samples [1]. People with PD are at increased risk of co-morbid health problems, substance misuse [2] and cardiovascular disease [3]. It is now well established that serious mental disorder is associated with early mortality [4]. However, only very recently has it emerged that individuals with a PD diagnosis also have substantially reduced life expectancy [5],[6], with increased mortality from both natural and unnatural causes [6], [7], [8]. The excess mortality risks are particularly high for younger people with personality disorder [5]. Yet, within the population of individuals with PD, little is known about the clinical predictors of natural and unnatural mortality. Premature death in people with PD may arise as a result of a number of mechanisms. For example, people with PD often have difficulty with emotional regulation, which they may try to manage with behaviours carrying significant health risks, such as self-harm, and alcohol and substance abuse. These same behaviours also carry a risk of accidental death. Comorbid axis-I psychopathology [9], [10], tendency to hostility and aggression [11], and poor psychosocial functioning are common features among individuals with PD and may partially account for the excess mortality, along with recognised associations between PD and poor health [3], [12]. However, these are speculative mechanisms with little empirical data to support or refute them.

No previous study has examined the independence of clinical risk factors for mortality among patients with PD. This is an important gap in the literature, as effective interventions to reduce mortality must be based on a thorough knowledge of the specific risk factors predicting mortality in the population in question. With this in mind, we set out to investigate the independence of a set of *a priori* clinical predictors for all-cause, natural and unnatural mortality, among individuals with PD known to secondary mental health services.

## Method

### Setting

Our sample was drawn from the electronic clinical records of the South London and Maudsley NHS Foundation Trust (SLAM). SLAM is a secondary mental health care provider that serves an aggregate population of 1.2 million people living in four London boroughs (Lambeth, Southwark, Lewisham and Croydon). Electronic clinical records have been used comprehensively across all SLAM services since 2006 and the SLAM Biomedical Research Centre (BRC) Clinical Record Interactive Search (CRIS) system was developed in 2008 to allow searching and retrieval of anonymised information from full clinical records with approximately 230,000 cases currently represented on the system. The development and protocol of CRIS has been described in detail [13], as has the process for case note anonymisation [14]. CRIS was approved as a data resource for secondary analysis by the Oxfordshire Research Ethics Committee (reference 08/H0606/71+5). As CRIS is an anonymised and de-identified database there is no requirement for individual participant consent for this study.

### Inclusion Criteria

The analysed cohort was extracted from the CRIS system and comprised all individuals meeting all of the following criteria:

- Age greater than 15 years;
- Primary International Classification of Diseases, 10^th^ Edition (ICD-10) [15] diagnosis of PD (categories F60 and F61) on case record within the period from 1 January 2007 to 31 December 2011;
- Assessed by a clinician using the Health of the Nations Outcome Scale (HoNOS) at least once during this same period.

The face validity of PD diagnoses on the CRIS system has been examined against blinded clinician rating of case note document, with a kappa coefficient of 0.72 (p<0.001) for level of agreement [16].

### Main outcome measures

We defined three five-year outcomes: all-cause mortality, natural and unnatural mortality. The beginning of 2007 was chosen as a starting point for the observation because this corresponded to the most complete recording of clinical data on the CRIS system.

#### Death identification

All death certifications are linked to NHS numbers. Every death in the UK, after the issuing of a formal death certificate, must be reported to the Office for National Statistics General Records Office and conveyed to the NHS Care Records Service, which holds these death notifications and makes them available to all NHS organisations. Accordingly, on a weekly basis, SLAM downloads a list of deceased patients from the NHS Care Records Service and updates their dates of death onto the patients' records, whether that person is active to services or has been discharged. In the present study, deaths determined by a date of death within the 5-year period were enrolled for analyses.

#### Cause of Death

Death certification data on all deaths in CRIS cases up to the end of 2011 was obtained from the Health & Social Care Information Centre (HSCIC). Cause of death data, in the form of ICD-10 codes, were matched to deceased cases in the study cohort using individual NHS number. Natural causes of death were defined as those with ICD-10 codes A00-R99 (major diagnostic categories), while unnatural causes were identified by ICD-10 codes V01-Y89, U509 (accidental, intentional, and undetermined).

In the case of a deceased individual not having corresponding cause of death data identified by this method, anonymised records were extracted using CRIS and manually scrutinised by a clinician (MF) for information pertaining to natural/unnatural cause of death.

### Explanatory variables

#### Demographic and socioeconomic factors

Date of birth, gender, and ethnicity were defined from routinely completed fields on the source records. Age was calculated from the patient's PD diagnosis date. Ethnicity classifications were: “White British or other white background”, “East Asian”, “South Asian”, “African, Caribbean or other black background”, and “Mixed, unknown, and others”.

The index of multiple deprivation is an area-level measure of socioeconomic status, calculated at the level of lower super output area for the residence (LSOA) – a UK address-grouping construct which contains a minimum of 1000 residents and 400 households, and an average of 1,500 residents. The index of multiple deprivation is derived from multiple domains including: employment, income, education, health, barriers to housing and services, crime and the living environment. Each domain is given a specific weighting to reflect its overall importance in the calculation of this index. Moreover, each domain is made up of a number of specific indicators that reflect different aspects of the deprivation they are intended to measure. Full details of each domain, the indicators they contain and the domain weightings that were used to derive the index of multiple deprivation are reported elsewhere [17]. In this study, a patient's residential postcode in England that was recorded closest in time to the beginning of the observation period was used to obtain an index of multiple deprivation score, which was used in the analysis as a proxy for socioeconomic status. Increasing scores in the index of multiple deprivation are indicative of more severe deprivation. In the analysis, deprivation scores were divided into tertiles. A separate category was given for homelessness.

#### Clinical variables

We rated the presence and severity of key clinical problems using the Health of the Nation Outcome Scale (HoNOS), a widely used and validated, 12-item, clinician-administered measure [18], [19], [20]; a review of the psychometric properties of the HoNOS by Pirkis et al found that the instrument had good validity and adequate reliability overall [19]. We selected the following 8 HoNOS items for investigation as potential risk factors for mortality, on *a priori* grounds: (1) overactive or aggression; (2) non-accidental self-injury; (3) problem-drinking or drug-taking; (5) physical illness or disability problems; (6) problems associated with hallucinations and delusions; (7) problems with depressed mood; (9) problems with social relationships; and (10) problems with activities of daily living (ADL) – the latter refers to problems with basic activities of self-care (e.g. eating, washing, dressing, toilet) as well as more complex skills such as budgeting, shopping, and use of transport. The eight exposures were chosen in order to represent a range of non-demographic variables (behavioural, co-morbid symptoms, health status and functioning status) that have been associated with adverse outcomes including mortality in previous studies investigating personality disorder or other mental disorders [21], [22], [23], [24], [25].

The HoNOS items have operationalized response options that follow the format of: 0 “not a problem”; 1 “subclinical, minor problem requiring no action”, 2 “mild problem but definitely present”, 3 “ moderately severe problem”, and 4 “severe to very severe problem” [26]. In this analysis, we used items from the first HoNOS questionnaire that was completed during the observation period as measures of baseline level of clinical severity in each patient. Due to small numbers in some categories, for the purposes of data analysis, all items were collapsed into four categories: 0) not a problem; 1) subclinical problem; 2) mild problem, and 3–4) severe or very severe problem.

### Statistical analysis

We used Cox proportional hazards regression to model the effect of the above risk factors on 1) all-cause mortality, 2) natural cause mortality and 3) unnatural cause mortality, respectively. For each patient the ‘at-risk’ period commenced from the date of the PD diagnosis. The censoring date was the end of the observation period (31st December 2011) for those who survived until the end of the observation period, and the event date was the date of death if this occurred during the observation period. Crude and adjusted associations between all-cause, natural cause and unnatural cause mortality and the principal exposures of interest (HoNOS subscale scores) or potential confounders were examined. HoNOS subscales that are associated with increased mortality risk were included in subsequent adjusted analyses. In the adjusted analyses, three levels of adjustment were used: the first model included only age and gender; the second model also included ethnicity and deprivation in area of residence (i.e. all demographic variables). The third and final model included all variables in the second model plus all HoNOS subscale ratings.

## Results

We identified 4296 cases of PD, of whom 2440 (56.8%) had at least one HoNOS rating in the observation period. Having at least one HoNOS was not associated with death within the observation period or with gender, but it was associated with older age [mean age (standard deviation) 38.2 (13.0) vs. 36.0 (12.9); p<0.001]. Therefore a total of 2,440 cases with PD formed the analysed cohort, of whom 85 (3.4%) died within the 5-year observation period. The mean follow-up period was 985.5 (SD 550.6) days. Of the 85 deaths, 16 required scrutiny of free-text data in order to classify natural or unnatural cause of death, which remained unknown in 6 cases. Of the 79 deaths with known cause, 49 (62%) were from natural causes and 30 (38%) were from unnatural causes. Table 1 displays number of cases and deaths from all causes by cohort characteristics, and unadjusted hazard ratios. Older age was associated with increased mortality risk, and African, Caribbean or other black ethnic group was associated with decreased risk. HoNOS subscales associated with increased mortality risk were overactivity / aggression, drinking / drug use, physical illness / disability, and problems with ADL. HoNOS subscales that were not associated with mortality risk were omitted from the subsequent adjusted models (Tables 2–4), with the exception of non-accidental injury, because self-injury is a prevalent problem in people with PD and is also a well-established predictor of mortality in previous studies [22], [27].

**Table 1:** Cohort characteristics and crude hazard ratios for association with all-cause mortality.

Variables	Number of individuals (Number of deaths)	% deaths	Crude Hazard Ratio (95% CI)
Total	2440 (85)		
**Gender**			
Female	1372 (42)	3.1	Referent
Male	1068 (43)	4.0	1.3 (0.8–1.9)
**Age group**			
15–29 years	763 (13)	1.7	Referent
30–44	976 (22)	2.3	1.1 (0.6–2.3)
45–64	610 (34)	5.6	3.0 (1.6–5.7)**
65+	91 (16)	17.6	12.0 (5.8–24.9)***
**Ethnicity**			
White British or other white	1764 (73)	4.1	Referent
East Asian	38 (3)	7.9	2.0 (0.6–6.5)
South Asian	35 (0)	0.0	–
African, Caribbean or other black	388 (7)	1.8	0.4 (0.2–0.9)*
Mixed/unknown	215 (2)	0.9	0.3 (0.1–1.1)
**Deprivation in area of residence**			
Low	741 (22)	3.0	Referent
Medium	749 (28)	3.7	1.3 (0.7–2.2)
High	749 (30)	4.0	1.4 (0.8–2.4)
Homeless/Missing/unknown	201 (5)	2.5	0.8 (0.3–2.0)
**Non-accidental self injury**			
Not a problem	1404 (51)	3.6	Referent
Subclinical, minor problem	389 (9)	2.3	0.7 (0.3–1.3)
Mild problem	311 (11)	3.5	1.1 (0.6–2.0)
Severe/very severe problem	325 (14)	4.3	1.4 (0.7–2.4)
Missing	11 (0)	0.0	
**Overactivity and aggression**			
Not a problem	1123 (30)	2.7	Referent
Subclinical, minor problem	659 (30)	4.6	1.7 (1.0–2.9)*
Mild problem	379 (15)	4.0	1.6 (0.8–2.9)
Severe/very severe problem	270 (10)	3.7	1.5 (0.7–3.0)
Missing	9(0)	0.0	
**Depressed mood**			
Not a problem	522 (18)	3.5	Referent
Subclinical, minor problem	638 (24)	3.8	1.1 (0.6–2.0)
Mild problem	785 (26)	3.3	1.0 (0.5–1.8)
Severe/very severe problem	484 (17)	3.5	1.1 (0.6–2.1)
Missing	11 (0)	0.0	
**Hallucinations and delusions**			
Not a problem	1674 (56)	3.4	Referent
Subclinical, minor problem	310 (7)	2.3	0.6 (0.3–1.4)
Mild problem	250 (10)	4.0	1.2 (0.6–2.3)
Severe/very severe problem	193 (12)	6.2	1.7 (0.9–3.2)
Missing	13 (0)	0.0	
**Drinking or drug use**			
Not a problem	1447 (44)	3.0	Referent
Subclinical, minor problem	305 (5)	1.6	0.6 (0.2–1.4)
Mild problem	298 (15)	5.0	1.7 (0.9–3.0)
Severe/very severe problem	363 (21)	5.8	1.9 (1.1–3.2)*
Missing	27 (0)	0.0	
**Physical illness or disability**			
Not a problem	1484 (29)	2.0	Referent
Subclinical, minor problem	363 (14)	3.9	2.0 (1.0–3.7)*
Mild problem	322 (15)	4.7	2.2 (1.2–4.1)*
Severe/very severe problem	253 (27)	10.7	5.5 (3.3–9.4)**
Missing	18 (0)	0.0	
**Relationships**			
Not a problem	563 (19)	3.4	Referent
Subclinical, minor problem	531 (18)	3.4	1.0 (0.5–1.9)
Mild problem	731 (21)	2.9	0.9 (0.5–1.6)
Severe/very severe problem	594 (26)	4.4	1.4 (0.8–2.6)
Missing	21 (1)	4.8	
**Activities of daily living**			
Not a problem	1179 (29)	2.5	Referent
Subclinical, minor problem	584 (16)	2.7	1.0 (0.6–1.9)
Mild problem	453 (20)	4.4	1.7 (1.0–3.0)
Severe/very severe problem	200 (20)	10.0	4.2 (2.4–7.4)***
Missing	24 (0)	0.0	

*p<0.05;

**p<0.01;

***p<0.001.

**Table 2:** Cox regression analyses of factors associated with all-cause mortality amongst individuals with personality disorder.

Risk Factors	Hazard Ratio (95% CI)		
	Adjusted for age† and gender	Adjusted for all demographic^a^ factors	Fully adjusted^b^
**Non-accidental self injury**			
Not a problem	Referent	Referent	Referent
Subclinical, minor problem	0.8 (0.4–1.7)	0.8 (0.4–1.6)	0.7 (0.3–1.4)
Mild problem	1.5 (0.8–3.0)	1.5 (0.7–2.8)	1.0 (0.5–1.9)
Severe/very severe problem	2.1 (1.1–3.8)*	2.0 (1.1–3.8)*	1.3 (0.7–2.5)
**Overactivity and aggression**			
Not a problem	Referent	Referent	Referent
Subclinical, minor problem	1.7 (1.0–2.8)*	1.6 (1.0–2.7)	1.6 (1.0–2.7)
Mild problem	1.4 (0.7–2.5)	1.4 (0.7–2.5)	1.1 (0.6–2.1)
Severe/very severe problem	1.5 (0.7–3.0)	1.6 (0.8–3.2)	1.0 (0.5–2.1)
**Drinking or drug use**			
Not a problem	Referent	Referent	Referent
Subclinical, minor problem	0.7 (0.3–1.7)	0.7 (0.3–1.7)	0.6 (0.3–1.6)
Mild problem	2.4 (1.3–4.3)**	2.3 (1.3–4.2)**	2.1 (1.1–3.9)*
Severe/very severe problem	2.7 (1.6–4.6)***	2.8 (1.6–4.7)***	2.3 (1.3–4.1)**
**Physical illness or disability**			
Not a problem	Referent	Referent	Referent
Subclinical, minor problem	1.4 (0.7–2.7)	1.4 (0.7–2.6)	1.3 (0.7–2.5)
Mild problem	1.4 (0.7–2.6)	1.3 (0.7–2.5)	1.2 (0.6–2.3)
Severe/very severe problem	3.0 (1.7–5.3)***	2.8 (1.6–5.0)***	1.9 (1.0–3.6)*
**Activities of daily living**			
Not a problem	Referent	Referent	Referent
Subclinical, minor problem	0.9 (0.5–1.7)	0.9 (0.5–1.7)	0.9 (0.5–1.6)
Mild problem	1.5 (0.8–2.6)	1.5 (0.9–2.7)	1.2 (0.7–2.1)
Severe/very severe problem	2.7 (1.5–4.8)**	2.8 (1.5–5.0)**	1.9 (1.0–3.6)*

† Entered as a continuous variable in all models.

a Demographic factors = age, gender, ethnicity, deprivation.

b Adjusted for demographic factors and all other variables that appear in this table.

*p<0.05;

**p<0.01;

***p<0.001.

**Table 3:** Cox regression analyses of factors associated with natural cause mortality amongst individuals with personality disorder.

Risk Factors	Number of individuals (Number of natural deaths)	Hazard Ratio (95%CI)		
		Adjusted for age† and gender	Adjusted for all demographic^a^ factors	Fully adjusted^b^
**Non-accidental self injury**				
Not a problem	1404 (33)	Referent	Referent	Referent
Subclinical, minor problem	389 (4)	0.7 (0.2–1.9)	0.7 (0.2–1.9)	0.7 (0.2–2.1)
Mild problem	311 (8)	2.2 (1.0–4.9)	2.2 (1.0–5.0)	1.4 (0.6–3.3)
Severe/very severe problem	325 (4)	1.2 (0.4–3.4)	1.2 (0.4–3.6)	0.7 (0.2–2.3)
**Overactivity and aggression**				
Not a problem	1123 (18)	Referent	Referent	Referent
Subclinical, minor problem	659 (14)	1.3 (0.7–2.7)	1.3 (0.6–2.5)	1.2 (0.6–2.4)
Mild problem	379 (11)	1.3 (0.6–2.9)	1.3 (0.6–2.9)	0.9 (0.4–2.0)
Severe/very severe problem	270 (6)	1.4 (0.6–3.6)	1.6 (0.6–4.1)	1.0 (0.4–2.7)
**Drinking or drug use**				
Not a problem	1447 (26)	Referent	Referent	Referent
Subclinical, minor problem	305 (4)	1.1 (0.4–3.1)	1.0 (0.4–3.0)	1.0 (0.3–3.0)
Mild problem	298 (10)	3.8 (1.8–8.3)**	3.8 (1.7–8.4)**	3.4 (1.5–7.4)**
Severe/very severe problem	363 (9)	2.9 (1.3–6.4)*	2.9 (1.3–6.7)*	2.4 (1.0–5.8)
**Physical illness or disability**				
Not a problem	1484 (12)	Referent	Referent	Referent
Subclinical, minor problem	363 (10)	1.8 (0.8–4.3)	1.8 (0.7–4.3)	1.8 (0.8–4.4)
Mild problem	322 (6)	0.9 (0.3–2.6)	0.8 (0.3–2.3)	0.8 (0.3–2.2)
Severe/very severe problem	253 (21)	3.7 (1.7–8.0)**	3.5 (1.6–7.7)**	2.4 (1.0–5.6)*
**Activities of daily living**				
Not a problem	1179 (15)	Referent	Referent	Referent
Subclinical, minor problem	584 (7)	0.7 (0.3–1.8)	0.7 (0.3–1.7)	0.7 (0.3–1.6)
Mild problem	453 (11)	1.4 (0.6–3.1)	1.4 (0.7–3.2)	1.1 (0.5–2.4)
Severe/very severe problem	200 (16)	3.0 (1.4–6.3)**	3.2 (1.5–6.8)**	2.2 (0.9–4.9)

† Entered as a continuous variable in all models.

a Demographic factors = age, gender, ethnicity, deprivation.

b Adjusted for demographic factors and all other variables that appear in this table.

*p<0.05;

**p<0.01;

***p<0.001.

**Table 4:** Cox regression analyses of factors associated with unnatural cause mortality amongst individuals with personality disorder.

Risk Factors	Number of individuals (Number of unnatural deaths)	Hazard Ratio (95%CI)		
		Adjusted for age† and gender	Adjusted for all demographic^a^ factors	Fully adjusted^b^
**Non-accidental self injury**				
Not a problem	1404 (15)	Referent	Referent	Referent
Subclinical, minor problem	389 (5)	1.3 (0.5–3.5)	1.2 (0.4–3.3)	1.0 (0.3–2.8)
Mild problem	311 (3)	1.0 (0.3–3.6)	0.9 (0.3–3.2)	0.7 (0.2–2.7)
Severe/very severe problem	325 (7)	2.5 (1.0–6.2)	2.3 (0.9–5.8)	1.5 (0.6–4.1)
**Overactivity and aggression**				
Not a problem	1123 (11)	Referent	Referent	Referent
Subclinical, minor problem	659 (12)	1.9 (0.8–4.2)	1.9 (0.8–4.3)	1.8 (0.8–4.1)
Mild problem	379 (3)	0.8 (0.2–3.0)	0.9 (0.2–3.2)	0.8 (0.2–3.0)
Severe/very severe problem	270 (4)	1.6 (0.5–5.0)	1.5 (0.5–4.8)	1.0 (0.3–3.5)
**Drinking or drug use**				
Not a problem	1447 (14)	Referent	Referent	Referent
Subclinical, minor problem	305 (1)	0.4 (0.0–2.7)	0.3 (0.0–2.5)	0.3 (0.0–2.5)
Mild problem	298 (4)	1.4 (0.5–4.4)	1.3 (0.4–4.0)	1.3 (0.4–4.1)
Severe/very severe problem	363 (11)	3.2 (1.4–7.1)**	3.2 (1.4–7.1)**	3.1 (1.3–7.3)*
**Physical illness or disability**				
Not a problem	1484 (15)	Referent	Referent	Referent
Subclinical, minor problem	363 (3)	0.8 (0.2–2.9)	0.8 (0.2–2.8)	0.7 (0.2–2.5)
Mild problem	322 (8)	2.4 (1.0–5.9)	2.4 (1.0–2.8)	2.1 (0.9–5.3)
Severe/very severe problem	253 (4)	1.7 (0.5–5.2)	1.5 (0.5–4.7)	1.1 (0.3–3.7)
**Activities of daily living**				
Not a problem	1179 (12)	Referent	Referent	Referent
Subclinical, minor problem	584 (8)	1.3 (0.5–3.1)	1.3 (0.5–3.2)	1.2 (0.5–3.1)
Mild problem	453 (7)	1.5 (0.6–3.7)	1.5 (0.6–3.8)	1.2 (0.5–3.2)
Severe/very severe problem	200 (3)	1.5 (0.4–5.4)	1.5 (0.4–5.3)	1.2 (0.3–4.5)

† Entered as a continuous variable in all models.

a Demographic factors = age, gender, ethnicity, deprivation.

b Adjusted for demographic factors and all other variables that appear in this table.

*p<0.05;

**p<0.01;

***p<0.001.

### All-cause mortality – adjusted models

Tests of the proportional hazards assumption indicated there was no violation and thus it was appropriate to proceed with Cox regression modelling. Table 2 displays Cox regression analyses of associations between clinical variables and all-cause mortality at three levels of adjustment – 1) adjusted for age and gender; 2) adjusted for age, gender, ethnicity and deprivation in area of residence (i.e. all demographics); and 3) adjusted for all demographics and all HoNOS subscales other than the exposure in question. Age was entered as a continuous variable in the models. All-cause mortality was associated with drinking / drug use, physical illness / disability, and problems with ADL at the first two levels of adjustment. All these associations were attenuated in the fully adjusted model, but they remained statistically significant. An association between the non-accidental injury subscale and all-cause mortality was observed after the first level of adjustment (age and gender); however at subsequent levels of adjustment it was no longer significant. An association between overactivity / aggression and all-cause mortality was observed in the first two models, but was no longer significant after adjusting for other HoNOS subscales.

### Natural cause mortality – adjusted models

Table 3 summarises Cox regression models of factors associated with natural cause mortality. As in Table 2, three levels of adjustment are shown. The non-accidental injury and overactivity / aggression HoNOS subscales were not associated with natural cause mortality. Mild and severe drinking / drug use were both associated with natural cause mortality across the first two adjusted models; however in the fully adjusted model only mild drinking / drug use remained significant. Severe physical illness / disability was associated with natural cause mortality across all three models. Severe problems with ADL was associated with natural cause mortality in the first two models, but was not significant in the final model.

### Unnatural cause mortality – adjusted models

Table 4 displays Cox regression models examining factors associated with unnatural cause mortality. Only severe drinking / drug use was associated with this outcome, an association which was significant across all three models.

## Discussion

In this large clinical cohort of people with a diagnosis of personality disorder, monitored over a 5-year period, more than fifty percent of deaths were accounted for by natural causes. Alcohol or drug use, physical illness, and impairment in ADL were all independently associated with all-cause mortality. Mortality from natural causes was independently associated with mild problems in alcohol or drug use, and severe physical illness, while unnatural cause mortality was predicted only by severe alcohol or drug use. Against our expectations, we did not find an association between the HoNOS subscale assessing non-accidental self-injury and any mortality outcome.

No previous research has investigated clinical predictors of either all-cause or cause-specific mortality in individuals with PD. Mortality studies in PD have instead almost exclusively investigated deaths from unnatural causes, particularly within borderline PD [28], . In borderline PD, depression, substance use disorder and antisocial PD (or traits) are associated with higher risk of completed suicide [28], [29]. However, despite the increased recognition of natural causes underlying excess mortality in people with mental disorders [30], [31], [32], no previous study has investigated deaths from natural causes among people with PD.

The recent Nordic psychiatric case register study by Nordentoft et al found that, in a cohort of over 270,000 patients with diagnoses of schizophrenia spectrum disorders, affective disorders, substance abuse or personality disorder, those with substance abuse or personality disorder had the most reduced life expectancy compared to the general population [6]. This chimes with the findings of previous mortality studies in psychiatric populations [7], [8], [33], [34]. Both substance abuse and PD are associated with deaths from diseases and medical conditions (i.e. natural causes) and with deaths from suicides, accidents and homicides (i.e. unnatural causes) [6], [33]. In our cohort of patients with PD, we found that higher scores on the HoNOS subscale assessing alcohol or drug use was associated with a two- to three-fold increased risk of death (both natural and unnatural). Deaths from accidents, homicides and suicides (i.e. unnatural causes) in patients abusing alcohol and/or illicit drugs might be explained by greater impulsivity, their involvement in a violent subculture or other risk behaviours. Considering natural causes of death, alcoholism is strongly linked with gastrointestinal disease, chiefly cirrhosis and peptic ulceration, whilst drug abuse is associated with viral infections, particularly hepatitis and HIV. It is noteworthy, however, that mild rather than severe alcohol or drug use predicted death from natural causes. One possible explanation for this finding is that substance use rated as mild in severity is more likely to go untreated. Similar mechanisms may help to explain an association between subclinical depression and mortality in patients with serious mental illness [35]. Another possibility is that those people with PD and severe alcohol or drug use who present to clinical services represent relatively healthy survivors, which would obscure any association with later mortality risk.

The detected association between all-cause and natural cause mortality with physical illness is unsurprising. PD is associated with poor health [12], and physical ill-health from unhealthy lifestyles, undertreated medical conditions and harmful side effects of medications are known to reduce life expectancy in people with mental disorders [36], [37]. Previous studies have reported substantially reduced life expectancy among individuals who self-harm [27], and frequency of self-harm is associated with increased risk of suicide [22]. In contrast, our study found no independent association between the HoNOS subscale on self-harm and mortality. Similarly, although high rates of violent behaviour in individuals with PD are a focus for clinical and public concern [1] and associations have been reported between hostility and mortality in cardiovascular disease [11], we found no association between overactivity and aggression, and mortality. On the other hand, difficulties with ADL independently predicted all-cause mortality. Together with the null findings with respect to self-harm and aggression, this is consistent with research showing that, in some cohorts, self-neglect may be a stronger predictor of mortality than more obvious risk factors such as suicide or violence [38]. It is also consistent with other research showing that ADL impairment is independently predictive of all-cause mortality among individuals with severe mental illness [24]. ADL impairment is therefore a potentially important marker of vulnerability in individuals with PD and further investigation is needed into the extent to which this is accounted for by poor psychosocial functioning and consequent chronic social disadvantage through social isolation and unemployment.

To our knowledge, this is the first study to investigate clinical predictors of all-cause and cause-specific mortality in individuals with personality disorder. A key strength of the study was the use of a large, representative clinical cohort, covering a broad age range and patients accessing various points of secondary care (inpatient admission, community care or one-off emergency presentation), increasing generalisability to other secondary care settings. We examined a wide range of clinical variables as exposures of interest, and included important potential demographic and socioeconomic confounders. The mortality data were drawn from death certification which is a legal requirement across the UK; under-ascertainment of deaths is therefore likely to be very low and only deaths occurring outside the UK are likely to be missed. However, the findings need to be considered in the light of certain limitations. Some measurement error is possible among demographic, socioeconomic as well as clinical variables (i.e. HoNOS items) when using routinely collected case record data; however, we would expect that any measurement error would be essentially random, so unlikely to introduce bias. Although we accounted for a wide range of clinical and socio-demographic variables, there may be residual confounding. In particular, we did not include frequency/intensity of service contact or account for possible effects of pharmacological or psychosocial interventions. Level of service contact and interventions may have a bearing on symptoms and health [39], either positively or negatively, which can contribute to mortality risk. Duration of illness and smoking are further variables that were not accounted for. A further limitation is the lack of power for examining more specific causes of death. Finally, we acknowledge that a large number of people with PD do not present to mental health services and are either managed in primary care or within general medical services. Our findings therefore only apply to secondary mental health service users.

Our findings are important and have clear implications for clinical practice. People with personality disorder are acknowledged to have reduced life expectancy [5], and this study has identified that the most risky subset of patients are those with alcohol and drug problems, poor physical health, and severe functional impairment. Each of these risk factors now demands attention.

The physical health status of patients with personality disorder should be regularly reviewed. We do not think that such a basic principle can be overstated, because we know that compared with members of the general population, people with mental health problems receive poorer physical healthcare [40]. Moreover, this problem is likely to be particularly pertinent to service users with a personality disorder, because they are often perceived to be ‘difficult’ [41] and not deserving of care [42]. Functional impairment is an enduring feature of most forms of personality disorder [43] and should therefore be a central component of the clinical assessment of people with suspected personality disorder. Finally, apparently mild problems with drugs and alcohol was the strongest predictor of mortality to emerge from our study, confirming the importance of taking an alcohol and drug history from personality-disordered patients, including those without conspicuous alcohol- and drug-related problems [35], [38].
